# Neuropsychiatric feature-based subgrouping reveals neural sensory processing spectrum in female FMR1 premutation carriers: A pilot study

**DOI:** 10.3389/fnint.2023.898215

**Published:** 2023-02-03

**Authors:** Jordan E. Norris, Lauren M. Schmitt, Lisa A. De Stefano, Ernest V. Pedapati, Craig A. Erickson, John A. Sweeney, Lauren E. Ethridge

**Affiliations:** ^1^Department of Psychology, The University of Oklahoma, Norman, OK, United States; ^2^Behavioral Medicine and Clinical Psychology, Cincinnati Children’s Hospital Medical Center, Cincinnati, OH, United States; ^3^Department of Pediatrics, University of Cincinnati, Cincinnati, OH, United States; ^4^Division of Child and Adolescent Psychiatry, Cincinnati Children’s Hospital Medical Center, Cincinnati, OH, United States; ^5^Division of Child Neurology, Cincinnati Children’s Hospital Medical Center, Cincinnati, OH, United States; ^6^Department of Psychiatry and Behavioral Neuroscience, University of Cincinnati, Cincinnati, OH, United States; ^7^Department of Pediatrics, Section on Developmental and Behavioral Pediatrics, The University of Oklahoma Health Sciences Center, Oklahoma City, OK, United States

**Keywords:** sensory processing, neural oscillations and entrainment, FMR1 premutation carrier, clustering analysis, EEG

## Abstract

**Introduction:**

Fragile X Syndrome (FXS) is rare genetic condition characterized by a repeat expansion (CGG) in the Fragile X messenger ribonucleoprotein 1 (FMR1) gene where individuals with greater than 200 repeats are defined as full mutation. FXS clinical presentation often includes intellectual disability, and autism-like symptoms, including anxiety and sensory hypersensitivities. Individuals with 55 to <200 CGG repeats are said to have the FMR1 premutation, which is not associated with primary characteristics of the full mutation, but with an increased risk for anxiety, depression, and other affective conditions, as well as and impaired cognitive processing differences that vary in severity. Defining subgroups of premutation carriers based on distinct biological features may identify subgroups with varying levels of psychiatric, cognitive, and behavioral alterations.

**Methods:**

The current pilot study utilized 3 cluster subgroupings defined by previous k means cluster analysis on neuropsychiatric, cognitive, and resting EEG variables in order to examine basic sensory auditory chirp task-based EEG parameters from 33 females with the FMR1 premutation (ages 17–78).

**Results:**

Based on the predefined, neuropsychiatric three-cluster solution, premutation carriers with increased neuropsychiatric features and higher CGG repeat counts (cluster 1) showed decreased stimulus onset response, similar to previous ERP findings across a number of psychiatric disorders but opposite to findings in individuals with full mutation FXS. Premutation carriers with increased executive dysfunction and resting gamma power (cluster 2) exhibited decreased gamma phase locking to a chirp stimulus, similar to individuals with full mutation FXS. Cluster 3 members, who were relatively unaffected by psychiatric or cognitive symptoms, showed the most normative task-based EEG metrics.

**Discussion:**

Our findings suggest a spectrum of sensory processing characteristics present in subgroups of premutation carriers that have been previously understudied due to lack of overall group differences. Our findings also further validate the pre-defined clinical subgroups by supporting links between disturbances in well-defined neural pathways and behavioral alterations that may be informative for identifying the mechanisms supporting specific risk factors and divergent therapeutic needs in individuals with the FMR1 premutation.

## 1. Background

Fragile X Syndrome (FXS) is a trinucleotide repeat disorder affecting the X chromosome characterized by a cytosine-guanine-guanine (CGG) expansion of the *FMR1* gene (Verkerk et al., 1991; Yu et al., 1991; Ciaccio et al., 2017). FXS is the most common monogenic cause of intellectual disability and autism. Repeat expansions > 200 CGG repeats are referred to as full mutations. These expansions result in extensive methylation of the gene, large reductions in Fragile X protein (FXP or Fragile X Messenger Ribonucleoprotein (FMRP), and symptoms of FXS. Repeat expansions falling between a typical CGG repeat count and 200 repeats (i.e., 55 – 200 CGG repeats) on the *FMR1* gene result in a premutation, which is associated with increased *FMR1* mRNA accompanied by minor reduction in protein levels, but which is not associated with the full cognitive and behavioral presentation of FXS (Fu et al., 1991; Wheeler et al., 2014). While individuals with FXS typically presents with varying levels of sensory processing difficulties (i.e., sensory hypersensitivities), individuals with the *FMR1* premutation are less affected and demonstrate a different pattern of behavioral symptoms than the full mutation (Schmitt et al., 2019).

Individuals with the *FMR1* premutation (premutation carrier – PMC) exhibit considerable variability in the type and severity of their clinical profiles (Budimirovic et al., 2020). PMCs exhibit complex health profiles including two known conditions associated with aging: Fragile X Tremor Ataxia Syndrome (FXTAS) and Fragile X Primary Ovarian Insufficiency (FXPOI) (Berry-Kravis et al., 2007). Beyond FXTAS and FXPOI, PMCs present with increased rates of psychopathology risk that change across the lifespan. Children with the premutation present with more frequent diagnoses of Attention Deficient Hyperactivity Disorder (ADHD), autism spectrum disorder (ASD), anxiety, and social difficulties, whereas adult PMCs primarily present with increased rates of anxiety and depression, particularly in females, who are twice as likely to have a premutation status than males due to the location of the *FMR1* gene on the X chromosome (Hunter et al., 2014; Cordeiro et al., 2015; Hagerman et al., 2018). Neuropsychiatric features of the premutation based on patterns of psychiatric, health, and behavioral symptoms were recently classified as Fragile X Associated Neuropsychiatric Disorder (FXAND) signifying the prevalence of psychiatric symptoms among PMCs. Further, PMCs are at higher risk for exacerbation of underlying psychopathology by comorbidities associated with the premutation and external experiences (e.g., close relative with the full mutation) resulting in greater impact of the premutation on quality of life (Hall et al., 2016; Wheeler et al., 2017; Hagerman et al., 2018; Allen et al., 2020; Klusek et al., 2020). Female adult PMCs may experience impaired executive function and social processing difficulties in addition to increased neuropsychiatric risk (Schmitt et al., 2022). Executive dysfunction and social processing difficulties were previously noted in child PMCs, in addition to the developmental differences between PMCs and TD infants centered around abnormal sensory experiences and non-verbal communication (Wheeler et al., 2016).

Abnormal sensory processing is a common phenotypical feature of the FXS full mutation manifesting typically as sensory hyperreactivity (Ethridge et al., 2019). Few studies have addressed sensory sensitivity in PMCs and also fail to utilize measures of neural responses such as electroencephalography (EEG) to sensory stimulation (Hunter et al., 2008; Allen et al., 2020; Schmitt et al., 2022). Most prior evaluations focused on child PMCs from a developmental perspective, limiting commentary on how moderate *FMR1* expansions impact sensory processing capacity later in the lifespan. Previous studies in child PMCs have documented co-occurring sensory hyper- and hypo-responsivity that vary based on age (Wheeler et al., 2016; Raspa et al., 2018). Most studies of sensory processing abnormalities in adult PMCs focused on their relation to mood and anxiety (Allen et al., 2020; Schmitt et al., 2022). The neural basis of sensory processing alterations remain unclear, as is the mechanism by which alterations at the neural level are related to clinical presentation of neuropsychiatric symptoms in adult PMCs.

We recently addressed clinical profile variability in the neuropsychiatric, executive function, eye tracking, and resting EEG features of PMCs using cluster-based methods. Clustering successfully categorized PMCs into three distinct subgroups based on affective, cognitive, and behavioral features. A three-cluster solution neatly identified one group with increased neuropsychiatric features (cluster 1) including increased depression and anxiety, but relatively typical resting EEG features. The second cluster (cluster 2) was characterized by increased executive dysfunction and higher resting gamma power suggesting a group more comparable to full mutation FXS. Finally, the third cluster (cluster 3) was comprised of members who were relatively unaffected by psychiatric or cognitive symptoms (Schmitt et al., 2022). However, this initial study did not examine or utilize sensory self-report or sensory EEG (such as sensory evoked potentials) measures or EEG measures directly related to mood and anxiety (e.g., frontal asymmetry) in deriving the cluster solution. As changes in sensory function measured both by self/caregiver report and sensory EEG are highly prevalent in full mutation FXS (Ethridge et al., 2019), it is of interest to examine these features, their distribution, and relevance to more commonly reported neuropsychiatric features in the FXS premutation. Frontal asymmetry measures have previously been linked to variations in mood and anxiety (Allen et al., 2004; Coan and Allen, 2004; Thibodeau et al., 2006; Stewart et al., 2010; Smith et al., 2017; van der Vinne et al., 2017), relevant to individuals with the FXS premutation. Changes in frontal asymmetry have also been reported in full mutation FXS, related to sensory processing and adaptive behavior (Norris et al., 2022). Use of predefined clustering may provide insight into the neural basis of sensory, mood, and anxiety processing in adult PMCs specific to the features that defined each cluster and provide defined neural pathways and behavioral alterations associated with varying clinical profiles. In this sense, any differences in sensory or asymmetry EEG measures between clusters serves as an external validation of cluster results, in that these measures were not used to define initial clusters but rather support additional sources of variation linked to cluster-defining features of PMCs.

The current pilot study aimed to extend the work detailed in Schmitt et al. (2022) by evaluating cluster membership in relation to task-based EEG parameters obtained during an auditory task, considering dynamic evaluations of oscillatory frequency bands in order to test the hypothesis that neuropsychiatric features are present in PMC populations supported by similar neural processes observed in full mutation FXS. Although the current study should be considered a pilot study and hypothesis generating due to the relatively small sample size and sparse literature on EEG in PMCs, we opted to also evaluate cluster differences across clinical measures as they related to variations in task-related frontal hemispheric asymmetry during the auditory task, as frontal asymmetry measures have previously been linked to variations in mood and anxiety hypothesized to map onto similar characteristics in PMCs (Allen et al., 2004; Coan and Allen, 2004; Thibodeau et al., 2006; Stewart et al., 2010; Smith et al., 2017; van der Vinne et al., 2017).

## 2. Materials and methods

### 2.1. Participants

Participants were 33 adult females with the FMR1 premutation [Mean (M) age = 48.36, SD = 13.17; age range 19.61 – 78.36] (Table 1). Only female PMCs were recruited to control for biological sex and ensure feasible ascertainment. The PMC sample was a convenience sample recruited from primary relatives (i.e., parent, grandparent, or sibling) of individuals with FXS through the Cincinnati Fragile X Research and Treatment Center, to pilot test for differences in individuals with the FMR1 premutation on measures that show significant differences in individuals with the FMR1 full mutation. PMC status was confirmed via medical record review or by a previously completed PCR quantification of allele specific CGG repeat length. Eleven PMC were currently taking antidepressant (AD) medications, consistent with reported rates of AD use and recommendations for treating PMCs experiencing mood disorders (Cordeiro et al., 2015; Hagerman et al., 2018). Exclusion criteria included history of seizures and current use of anticonvulsant and benzodiazepine medications due to known EEG effects of both medications.

**TABLE 1:** Participant demographics.

	Premutation carriers (*N* = 33)					
	Cluster 1 (*N* = 8)		Cluster 2 (*N* = 10)		Cluster 3 (*N* = 11)	
General	M	SD	M	SD	M	SD
Age	46.73	17.19	50.47	14.54	51.79	8.92
CGG repeat count	107.33	11.61	93.11	14.63	96.11	20.84
**Clinical mood measures**						
ASI	24.38	6.32	18.29	9.88	11.22	7.66
BDI	22.25	5.95	10.56	10.11	5.80	4.16

Demographic variables for PMCs by cluster.

Clinical self-report assessments included the Adolescent/Adult Sensory Profile (Brown et al., 2001), Anxiety Depression and Mood Scale (ADAMS, Esbensen et al., 2003), Becks’ Depression Inventory (BDI, Beck et al., 1961), and the Anxiety Sensitivity Index (ASI, Reiss et al., 1986). All participants provided written informed consent prior to participation, as approved by the Cincinnati Children’s Hospital Institutional Review Board.

### 2.2. Procedure

Participants underwent dense-array EEG while listening passively to a chirp stimulus. The chirp stimulus was an amplitude modulated white noise burst carrier stimulus increasing in frequency from 0-100 Hz over the course of 2,000 ms. Chirp stimuli were presented 200 times with each trial separated by an intertrial period that alternated between 1,500 ms and 2,000 ms. Stimuli were delivered via headphones at 65 db SPL while the participants watched a silent movie, consistent with prior studies using individuals with FXS (Ethridge et al., 2016, 2017, 2019; Smith et al., 2021; Pedapati et al., 2022).

### 2.3. EEG recording and data reduction

**FIGURE 1:**
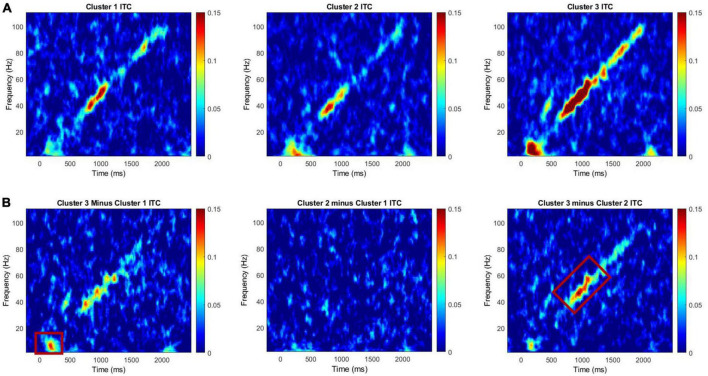
**(A)** Intertrial coherence for Clusters 1, 2, and 3 for the chirp task. **(B)** Difference plots for cluster 3 minus cluster 1, cluster 2 minus cluster 1, and cluster 3 minus cluster 2. Red boxes on difference maps indicate areas with significant group differences. Warmer colors in difference maps indicate higher phase-locking values for the first cluster and the cooler colors indicate higher values for the second cluster.

EEG recordings were collected using 128 channel hydrocel nets (Electrical Geodesics, Eugene, Oregon). EEG was recorded continuously and digitized at 1000 Hz, filtered from 0.01 to 200 Hz, referenced to Cz, and amplified 10,000x. Sensors were placed with landmark sensors according to the International 10/10 system (Chatrian et al., 1985; Luu and Ferree, 2005). Raw data were visually inspected offline. Bad sensors were interpolated using spherical spline interpolation (no more than 5% per subject for a total of 6 electrodes, no more than two adjacent, 96.97% of participants had no sensors interpolated within the 23 channels used to calculate general EEG variables for final analyses, 100% did not have F3 or F4 interpolated) implemented in BESA 6.1 (MEGIS Software, Grafelfing, Germany). Data were digitally filtered from 0.5 to 120 Hz (12 and 24 db/octave roll-off, respectively; zero-phase; 60 Hz notch) and data segments with large amounts of artifact were removed prior to individual component removal to facilitate algorithm convergence while running independent component analysis (ICA; Infomax). Lastly, large muscle artifacts (e.g., eye movement, cardiac, and muscle movement artifacts) were removed blind to participant group using ICA implemented in EEGLAB through Matlab 2018b software (Delorme and Makeig, 2004; The Mathworks, Natick, MA, United States). Data were transformed to average reference and epoched into 3,250 ms trials (-500 to 2,750 ms). Chirp task data were averaged across trials. Any trial with post-ICA amplitude exceeding 120 μV was considered residual artifact and removed prior to averaging (i.e., trials containing large quantities of movement artifact post-ICA were rejected). The same 23 electrodes were selected *a priori* for general level analysis from prior work with the FXS full mutation based on a fronto-central spatial distribution of the scalp best poised to capture auditory cortex activity (Luck, 2014; Ethridge et al., 2019). Intertrial coherence (ITC) and single trial power were calculated with un-baseline-corrected epoched single-trial data according to wavelet methods used previously for Ethridge et al. (2019) and using the same power bands across the entire epoch and ITC time-frequency clusters: low gamma ITC (30-55 Hz) during the chirp, high gamma ITC (65-80 Hz) during the chirp, chirp stimulus onset, and offset theta/alpha ITC (corresponding to time windows encompassing approximately 50-400 ms after chirp stimulus onset and again post- chirp stimulus offset (2,050-2,400 ms in Figure 1), and a 4-13 Hz frequency range from previous work. Single trial power evaluates the amplitude of the neural response whereas ITC evaluates consistency of phase of the neural response frequency at any given timepoint across trials (Delorme and Makeig, 2004).

### 2.4. Frontal asymmetry EEG analysis

Data were transformed to current source density (CSD)-Laplacian in BESA 6.1 for frontal asymmetry analyses and hemisphere specific power analyses. CSD computation reduces volume conduction effects (i.e., signal smearing) and improves both temporal and spatial resolution (Stewart et al., 2010; Burle et al., 2015; Smith et al., 2017). Data from one electrode in each hemisphere were used to calculate frontal asymmetry scores according to conventional frontal asymmetry analysis methods (Allen et al., 2004; Stewart et al., 2010). Electrode F3 (left hemisphere) and electrode F4 (right hemisphere) were selected for data extraction and asymmetry was operationalized as (R – L)/(R + L) to compute a normalized difference score (NDS) that normalizes for overall power. The correlation between NDS and the natural log difference scores [i.e., ln(R)-ln(L)] was checked to confirm linearity with NDSs and found to correlate highly (rho > 0.9, *p* < 0.001) with the natural log calculation, as expected (Allen et al., 2004). Single trial power was extracted from each electrode to compute asymmetry in the theta (4-7 Hz), alpha (8-13 Hz), and low gamma (30-55 Hz) ranges for all recordings. All variables calculated using F3 and F4 are referenced as F3/F4 frontal EEG variables (e.g., F3/F4 frontal hemispheric power) to differentiate from power measures calculated using the initial 23 electrodes from previous work, referenced as general frontal power.

### 2.5. Statistical analysis

All statistical analyses were conducted in SPSS 27. Multivariate general linear models were utilized to assess hypothesis driven differences across clusters on EEG variables and clinical measures assessing sensory processing. Cluster membership was obtained from Schmitt et al. (2022), based on a k-means cluster analysis of the same participants using neuropsychiatric, executive functioning, social attention, eye-tracking measures, and resting EEG features. Effect sizes are reported as partial eta squared. All analyses included age as a covariate, retained where significant. All cluster comparisons were hypothesis driven. Clinical correlations were examined with all EEG variables. Exploratory correlations were also evaluated between power in frequency bands of interest (i.e., theta, alpha, and gamma) and hypothesis-driven associations with gamma ITC from FXS findings (Ethridge et al., 2019). All correlations were bivariate and conducted using Spearman’s rho. Clinical correlations and power band correlations were considered to be exploratory and hypothesis generating due to the current limited scope of neuropsychiatric research with PMC populations, and thus not corrected for multiple comparisons.

## 3. Results

### 3.1. Cluster differences on EEG measures

#### 3.1.1. ITC and power differences between clusters

**FIGURE 2:**
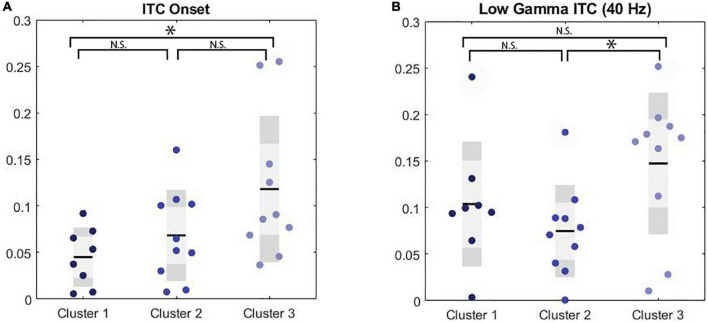
Significance markers reflect non-corrected MANCOVA results. N.S. = not significant, *p < 0.05. **(A)** Boxplot depicting group comparison across clusters for ITC to the onset of the chirp stimulus showed overall significance (*p* = 0.04) where cluster 1 showed a trend toward significantly lower ITC than cluster 3 (*p* = 0.05). **(B)** Boxplot depicting group comparison across clusters for low gamma ITC with significant overall (*p* = 0.04) where cluster 2 showed significantly lower ITC than cluster 3 (*p* = 0.03).

A MANCOVA was conducted to evaluate both general EEG variables (i.e., ITC to stimulus onset, ITC to stimulus offset, low gamma ITC to chirp, high gamma ITC to chirp, theta power, alpha power, gamma power) and F3/F4 frontal asymmetry variables across frequency bands (i.e., alpha, theta, and gamma) by PMC cluster membership. Age was a significant covariate for ITC in the low gamma range, only. ITC to the onset of the chirp stimulus was significantly different between clusters, *F*(2, 24) = 3.61, *p* = 0.043, ES = 0.23. *Post hoc* Bonferroni corrected comparisons identified trending differences on ITC onset between Cluster 3 (M = 0.15, SE = 0.02) and Cluster 1 (M = 0.05, SE = 0.02). ITC in the low gamma range was also significantly different between clusters with cluster 2 (M = 0.07, SE = 0.05) exhibiting reduced ITC compared to cluster 3 (M = 0.15, SE = 0.07), *F*(2, 24) = 3.76, *p* = 0.038, ES = 0.24. Lastly, there was a trending difference between clusters on frontal low gamma asymmetry with cluster 2 (M = 0.06, SE = 0.09) exhibiting trending increases in frontal low gamma asymmetry compared to cluster 1 (M = –0.03, SE = 0.09) *F*(2, 24) = 3.01, *p* = 0.068, ES = 0.20. None of the comparisons between clusters on power variables were significant; cluster differences were primarily in ITC. ITC reflects the ability to reliably reproduce neural oscillations across multiple trials, and in the case of the chirp stimulus, to reliably reproduce the oscillatory properties of the stimulus in time. (See Table 2 for univariate results including non-significant comparisons, Figure 1 for ITC plots and Figure 2 for box plots with individual data points).

**TABLE 2:** Univariate results for EEG data by cluster MANCOVA.

EEG variable	*F*(2,24)	*P* value	Effect size (Partial eta squared)
ITC stimulus onset	3.61	0.043	0.231
ITC stimulus offset	1.69	0.206	0.123
ITC low gamma	3.76	0.038	0.239
ITC high gamma	2.07	0.148	0.147
Theta power	1.33	0.283	0.100
Gamma power	0.256	0.776	0.021
Theta power asymmetry	1.56	0.231	0.115
Alpha power asymmetry	1.56	0.229	0.116
Gamma power asymmetry	3.01	0.068	0.200

See section “3.1. Cluster differences on EEG measures” of the text for Bonferroni-corrected individual cluster comparisons for significant univariate tests.

### 3.2. Cluster differences on clinical measures

Results for anxiety and depression scale scores by cluster are reported in Schmitt et al. (2022).

**FIGURE 3:**
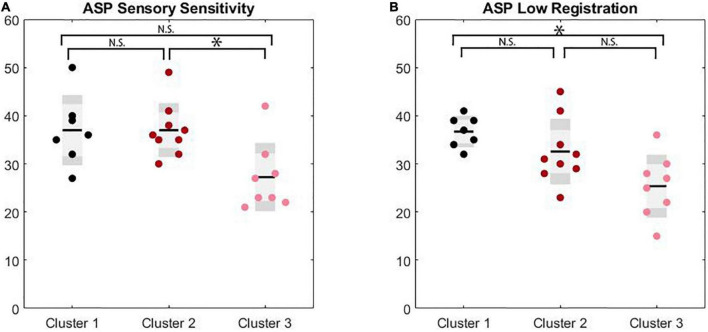
Significance markers reflect non-corrected MANCOVA results. N.S. = not significant, *p < 0.05. **(A)** Box plots depicting group comparison across clusters for ASP sensory sensitivity with overall significance (*p* = 0.02) where cluster 2 showed significantly higher sensitivity scores than cluster 3 (*p* = 0.02). **(B)** Box plots depicting group comparison across clusters for ASP low registration with overall significance (*p* = 0.03) where cluster 1 showed a trend toward significantly higher low registration scores than cluster 3 (*p* = 0.07).

A MANCOVA was conducted examining cluster differences across Adolescent/Adult Sensory Profile sub-scores to evaluate self-reported sensory experiences across clusters with differing clinical profiles. A significant difference was found on low registration (*F*(2,17) = 4.30, *p* = 0.031, ES = 0.34) and sensation sensitivity scores (*F*(2,17) = 5.06, *p* = 0.019, ES = 0.37) with differences on touch processing trending toward significance (*F*(2,17) = 3.43, *p* = 0.056, ES = 0.29). After Bonferroni correction, low registration differences between clusters were trending with Cluster 3 (M = 25.38, SD = 6.46) exhibiting lower scores compared to Cluster 1 (M = 35.00, SD = 2.94) and Cluster 2 (M = 32.56, SD = 6.73). Sensory sensitivity scores were significantly different between Cluster 2 (M = 37.00, SD = 5.52) and Cluster 3 (M = 27.25, SD = 7.01), with Cluster 2 exhibiting increased scores compared to Cluster 3. Cluster 1 showed intermediate sensory sensitivity scores (M = 34.50, SD = 6.14) and did not differ from either Cluster 2 or 3. Touch processing scores trended toward increases in Cluster 2 (M = 39.22, SD = 7.82) compared to Cluster 3 (M = 31.75, SD = 3.28), which Cluster 1 again showing an intermediate score (M = 33.50, SD = 4.51). (See Table 3 for full univariate results including non-significant comparisons, and Figure 3).

**TABLE 3:** Univariate results for sensory clinical data by cluster MANCOVA.

Sensory variable	*F*(2,17)	*P* value	Effect size (Partial eta squared)
Auditory processing	2.02	0.163	0.192
Low registration	4.30	0.031	0.336
Sensation seeking	0.352	0.708	0.040
Sensation sensitivity	5.06	0.019	0.373
Sensation avoiding	1.562	0.238	0.155
Touch processing	3.43	0.056	0.287

See section “3.2. Cluster differences on clinical measures” of the text for Bonferroni-corrected individual cluster comparisons for significant univariate tests.

### 3.3. Correlations across the PMC sample

Correlation analyses reflected an exploratory evaluation of EEG variables and clinical measures. Significant correlations for PMCs between general frontal EEG, F3/F4 frontal EEG, and clinical variables are shown in Table 4. General physiological correlations are presented in Table 5 to provide a broad assessment of the PMC neural phenotype across relationships generated from full mutation EEG findings. General frontal gamma power was correlated with all F3/F4 EEG measures across the alpha, theta, and low gamma frequency bands demonstrating unilateral use of high and low frequency bands. Power measures at the hemisphere level also demonstrated a pattern suggesting right hemisphere gamma was more strongly correlated with general frontal gamma, but this pattern was not significant, *z*(32) = −0.79, *p* = 0.21.

**TABLE 4:** Correlations between general frontal EEG variables, F3/F4 frontal EEG variables, and clinical variables for PMCs.

	General frontal EEG variables					
F3/F4 frontal EEG variables (*N* = 32)	Gamma power	Theta power	ITC stimulus onset	ITC stimulus offset	ITC low gamma	ITC high gamma
Left alpha power	0.41*	0.62**	-0.22	-0.15	0.03	-0.24
Right alpha power	0.62**	0.63**	-0.13	-0.15	-0.05	-0.38*
Left theta power	0.41*	0.59**	-0.25	-0.09	0.04	-0.21
Right theta power	0.57**	0.50**	-0.09	-0.08	-0.04	-0.31
Left low gamma power	0.67**	0.41*	0.03	0.18	-0.05	-0.22
Right low gamma power	0.75**	0.21	0.06	-0.01	-0.08	-0.31
Frontal alpha power asymmetry	0.05	-0.10	0.09	0.03	-0.12	-0.15
Frontal theta power asymmetry	0.004	-0.09	0.17	0.08	0.01	-0.05
Frontal low gamma power asymmetry	0.06	-0.12	0.23	0.03	-0.01	-0.005
**Clinical variables**						
BDI (*N* = 30)	-0.08	0.16	-0.46*	-0.41*	-0.19	-0.09
ASI (*N* = 27)	-0.05	0.15	-0.26	-0.21	-0.21	-0.19
ADAMs (*N* = 9)						
Manic/Hyperactive behavior	-0.10	-0.92**	-0.31	-0.35	-0.44	0.08
Depressed mood	0.19	-0.68*	-0.39	-0.18	-0.35	-0.30
Social avoidance	-0.07	-0.60	-0.24	-0.26	-0.24	0.18
General anxiety	0.08	-0.90**	-0.44	-0.31	-0.42	-0.11
Obsessive/Compulsive behavior	0.24	-0.67*	-0.71*	-0.44	-0.84**	-0.30
**ASP (*N* = 27)**						
Auditory processing	-0.03	-0.27	0.14	0.03	0.04	0.19
Taste/Smell processing	0.01	0.03	0.11	0.39	0.29	-0.02
Low registration	-0.33	-0.07	−0.37 (*p* = 0.058)	-0.25	-0.02	-0.03
Movement processing	0.20	-0.16	0.02	-0.25	-0.46*	-0.39
Visual processing	-0.05	0.11	-0.25	0.21	-0.04	-0.02
Touch processing	-0.03	-0.18	0.23	0.14	-0.15	0.11
Activity level	0.35	-0.11	0.08	0.35	-0.05	-0.07
Sensation seeking	-0.31	-0.19	-0.12	0.03	0.13	0.11
Sensation sensitivity	-0.24	-0.19	-0.02	0.19	-0.05	-0.11
Sensation avoiding	0.09	-0.08	-0.06	0.22	-0.19	-0.33

All correlations are Spearman’s rho and reflect all PMC across clusters. No asterisk = N.S. *p < 0.05, **p < 0.01.

**TABLE 5:** Correlations between General EEG Variables for PMCs.

	General EEG variables					
EEG variables	Gamma power	Theta power	ITC Stimulus onset	ITC Stimulus offset	ITC low gamma	ITC high gamma
Gamma Power	−	0.48**	-0.18	-0.07	-0.17	-0.49**
Theta Power		−	-0.12	0.001	0.19	-0.10
ITC Stimulus Onset			−	0.52**	0.49**	0.47**
ITC Stimulus Offset				−	0.27	0.24
ITC Low Gamma					−	0.64**
ITC High Gamma						−

All correlations are Spearman’s rho and reflect all PMCs across clusters. No asterisk = N.S. *p < 0.05, **p < 0.01.

Correlations between BDI scores and neural responses to the onset of the chirp stimulus adds support to the conclusion that hypo-responsivity shares a significant relationship with increased symptoms of depression. Increased general anxiety on the ADAMs also was associated with decreased responses to the onset of the chirp stimulus, but not decreased mood scores. Only one correlation was retained as significant between F3/F4 frontal EEG variables and clinical variables (not shown in Table 4): right alpha power was inversely correlated with manic/hyperactive behavior scores on the ADAMs (rho = −0.77, *p* = 0.015).

## 4. Discussion

The current study aimed to extend the understanding of *FMR1* PMC subgroupings established in a recent exploratory, data-driven approach that identified discrete clinical subgroups of PMC individuals (Schmitt et al., 2022) that varied in protein levels (FXP), CGG repeat length, environmental factors, and aging-associated outcomes (e.g., increased mRNA toxicity). We found that PMC with increased neuropsychiatric features and higher CGG repeat counts (Cluster (1) showed decreased stimulus onset response, similar to previous ERP findings across a number of psychiatric disorders (Drake et al., 1991; Ford et al., 2001; Ethridge et al., 2015) but opposite to findings in individuals with full mutation FXS (Ethridge et al., 2016, 2019). Premutation carriers with increased executive dysfunction and resting gamma power (Cluster 2) exhibited decreased gamma phase locking to a chirp stimulus, similar to individuals with full mutation FXS. Cluster 3 members, who were relatively unaffected by psychiatric or cognitive symptoms, showed the most normative task-based EEG metrics.

### 4.1. Neurophysiological differences by cluster

Differences among PMC subgroups suggest relevant sensory processing outcomes. Female PMCs in Cluster 1 (the “psychiatric” subgroup) showed the greatest reduction in neural response to stimulus onset relative to other clusters. The simplest interpretation of reduced stimulus onset response is sensory hyposensitivity, which may be linked to general neural hypo-responsivity and/or perceptual disturbances (Donkers et al., 2015). Clinically, Cluster 1 is comprised of individuals with affective disturbances (i.e., elevated depression and anxiety symptoms or the presence of symptoms related to affective disorders like depression or anxiety), suggesting that sensory hypo-responsivity may contribute to and/or co-occur with neuropsychiatric features for these individuals. This is a clinically relevant finding in that sensory systems present a novel target for therapy in PMC that may also have broader-ranging effects on mood and anxiety. Significant correlations between neural response to stimulus onset and clinical variables indicate PMCs may have increased sensory detection thresholds, which may lead to symptoms such as withdrawal, lethargy, and disinterest (i.e., low registration). Cluster 1 exhibiting higher low registration scores further supports this conclusion. Sensory hyposensitivity may mirror symptoms of depression or possibly render PMCs vulnerable to heightened self-reflection and internalizing behaviors (Kotsiris et al., 2020). Previous findings in individuals with depression have indicated a complex relationship between depressive symptoms and sensory processing, with individuals endorsing higher levels of sensory hypersensitivity *and* hyposensitivity (low registration) than controls (Engel-Yeger et al., 2016). Correlations between the neural response to onset and offset of the stimulus and BDI scores is consistent with the interpretation that sensory hypo-responsivity may contribute to risk or onset of affective disturbances (i.e., Cluster 1). Interestingly, individuals with full mutation FXS show increased neural response and hyperresponsivity to sensory stimuli, the opposite of these PMC findings, highlighting the differences in underlying pathophysiology between the two conditions (Ethridge et al., 2017, 2019).

Cluster 2 (the “executive dysfunction and atypical resting electrophysiology” subgroup) also showed reduced neural response to stimulus onset, and additionally showed reduced ITC to the chirp stimulus in the low gamma range. Cluster 2 also exhibited increased sensory sensitivity compared to other subgroups suggesting heightened sensory detection. Based on previous clustering results (Schmitt et al., 2022), Cluster 2 was comprised of individuals with symptoms of executive dysfunction and some resting EEG abnormalities. In particular, Cluster 2 showed reduced theta band resting power compared to typically developing controls, and theta power within Cluster 2 was associated with social processing in the opposite direction to the less affected Cluster 3, who showed increased theta power. Cluster 2 may exhibit reduced ability to mobilize compensatory activity in the theta range (Wang et al., 2017; Schmitt et al., 2022), which in this case may exacerbate sensory sensitivity symptoms. Indeed, the combination of reduced response to the onset of the chirp but comparably typical low gamma ITC in Cluster 1 suggests recovery in subsequent sustained high frequency sensory processing capacity, which is notably absent in Cluster 2, and which may differentiate neural pathways contributing to neural compensation and resilience in PMC. Individuals with FXS also show reductions in low gamma ITC to the chirp, which has been consistently correlated with increased autistic features (Ethridge et al., 2017, 2019), consistent with its potential contribution to alterations in sensory processing, and social and executive function deficits.

Consistent with our previous study (Schmitt et al., 2022), Cluster 3 consisted of individuals who were largely unaffected with the exception of notable resting state differences. Resting EEG differences in this subgroup may represent compensatory responses that reduce risk for psychiatric, executive function, and sensory processing deficits, although further research will be necessary to confirm a causative role.

### 4.2. Links between frontal asymmetry, depression, and anxiety

Previous evidence in the psychiatric literature links changes in frontal asymmetry to anxiety and depression (Smith et al., 2017), and frontal asymmetry differences have been described in full mutation FXS (Norris et al., 2022). Frontal alpha asymmetry has shown high internal consistency and moderate test-retest reliability in populations with depression, particularly greater right hemisphere frontal activity (Allen et al., 2004; Coan and Allen, 2004; Thibodeau et al., 2006; Stewart et al., 2010; van der Vinne et al., 2017). The relationship between frontal asymmetry and anxiety is similarly characterized by greater right hemisphere frontal activity (Thibodeau et al., 2006; Mathersul et al., 2008). However, individual differences in frontal alpha asymmetry may be related to underlying pathophysiology contributing to depression symptoms (Smith et al., 2017; van der Vinne et al., 2017). Rather than reflecting stable physiological alteration, measures of asymmetry may index more dynamic utilization of oscillatory frequency bands between hemispheres uniquely impacted by the *FMR1* premutation, which appear to be related to affective disturbances (Schmitt et al., 2022).

By subdividing the heterogenous PMC group into more homogenous clusters, we hoped to recover a more direct relationship between frontal asymmetry abnormalities and depression and anxiety symptoms, particularly in Cluster 1 individuals who show increased BDI and ASI scores. We did not however find any significant differences among clusters for theta and alpha frontal asymmetry, and only a trending difference between clusters for gamma asymmetry. We also did not find any correlations between frontal asymmetry measures and BDI or ASI scores across clusters, suggesting that these relationships identified in other patient populations with affective illness may not hold or be less robust in PMCs. The lack of relationship between frontal asymmetry and measures of mood dysregulation in PMCs may even reflect differential contributions of pathophysiology and environment to depression risk in PMC, who are typically identified for research studies due to their relationship to individuals in their household with FXS and who may experience additional stressors related to caring for a relative with a complex neurodevelopmental disorder (e.g., maternal stress; Hunter et al., 2019). Coupled with recent findings linking *FMR1* CGG repeat count to abnormal cortisol responses to stress in female PMC who were also mothers to a child with FXS (Hong et al., 2021), the absence of frontal asymmetry findings may indicate differential pathways to depression and anxiety risk in PMCs.

In contrast to our frontal asymmetry data, we did find significant correlations between decreased theta/alpha ITC to stimulus onset and increased scores on the BDI across the entire PMC group, suggesting that for PMC there is a relationship between sensory processing hypo-responsivity and mood. Reduced ERP amplitude and reduced ITC to sensory stimuli have been found in other psychiatric and neurodevelopmental groups, namely individuals with bipolar disorder (Isomura et al., 2016), autism (De Stefano et al., 2019; Hornung et al., 2019; Seymour et al., 2020; Roberts et al., 2021), and psychosis (Krishnan et al., 2009; Sugiyama et al., 2021), consistent with the psychiatric implications of these specific ERP features in PMCs. Reductions in theta power also were associated with increased symptoms of anxiety and depression in individuals who completed the ADAMS, including obsessive compulsive symptoms, suggesting a potential broader contribution for theta power to limbic regulation. Although these findings must be verified with a larger sample, the strong correlations do suggest a clinically meaningful association between reduced theta power, a universal feature of the PMC group rather than unique to one cluster, and overall risk for mood symptoms. Indeed, individuals in Cluster 3, who showed the smallest reductions in task-related theta power, and enhanced theta power at rest (Schmitt et al., 2022), also showed the fewest psychiatric and behavioral symptoms. While theta power typically decreases with healthy cognitive aging, age was not a significant correlate in any analyses regarding theta power, suggesting decreases in theta may reflect a typical neural presentation of the *FMR1* premutation (Cummins and Finnigan, 2007; Cellier et al., 2021). Theta power findings may be related to known anatomical shifts to limbic circuitry mediated by FMRP/elevated *FMR1* mRNA thought to heighten risk for psychopathology (Cordeiro et al., 2015). In conjunction with clinical correlations, these findings raise questions about the mechanistic underpinnings of psychiatric symptoms. We interpret the correlation results between clinical measures of mood dysregulation and EEG measures as reflective of underlying neural processes related to depression and anxiety but acknowledge the inability to attribute causality. Correlations between theta and mood dysregulation are potentially reflective of (1) the effect of depression on EEG measures, or (2) antecedent/causal of depression. Theta power may therefore be a clinically relevant measure for evaluating mood and anxiety in PMC, as well as a target for assessing effect of therapeutics in advance of clinically meaningful behavioral changes.

### 4.5. Limitations and future research

Twelve participants reported current AD use (36.4%) suggesting a clinically relevant level of mood disturbance within the sample, though many clinical reports did not report a diagnostic history of depression. Use of AD medication was consistent across all clusters indicating successful treatment potentially impacted cluster membership (Schmitt et al., 2022). Standard measures of depression and anxiety, including the BDI and ASI, are self-report measures that potentially prompt participants to respond according to their current mood state (Beck et al., 1961). The ADAMS is another self-report measure of broad spectrum psychiatric conditions, including depressed mood and anxiety, that also potentially prompted current mood states rather than lifetime symptom experiences. Further, the ADAMs has not been used in PMC samples, and was only completed by a subset of the sample (*n* = 9), thus additional caution was taken with data interpretation. PMCs with a medical history of current AD use may also report fewer symptoms on the BDI due to response items on the BDI prompting reports of current mood disturbance (as reflective of their AD use) compared to their life history reports (Marques et al., 2013). Conclusions of reduced theta reflecting an underlying mechanism for depression and anxiety specific to PMCs is complicated by non-significant correlations between EEG measures and both BDI and ASI scores suggesting that reduced theta may reflect more lifetime risk than current depression symptoms. Current use of mood stabilizing medication may also explain EEG outcomes with BDI and ASI scores, as PMCs that reported current use of antidepressant medications (*N* = 11) likely exhibited reduced symptoms of mood dysregulation at the time of evaluation (Iosifescu, 2011). Our data does suggest however that the *FMR1* premutation, coupled with potential differences in life stressors, may create unique contributions to depression and anxiety outcomes; further evaluation of underlying mechanisms is necessary to provide more specific therapeutic interventions (Hagerman et al., 2018; Maltman et al., 2021; Schmitt et al., 2022).

The PMC sample was limited due to (1) the nature of the FMR1 premutation, and (2). the convenience nature of the sample. Primarily, PMCs were asked to participate if their child, sibling, or grandchild was participating in on-going FXS-specific research or were at the clinic for regular clinical care. Limited participant numbers per cluster impacted the capacity to attribute correlations strictly to individual clusters and correlations may reflect population parameters confounded by heterogenous premutation presentation. More research is required to parse the extent that EEG measures of decreased theta reflect a unique mechanism of depressed mood in PMC. This study was initially conducted as a pilot to determine whether task-based EEG features related to clinical variation could be identified in PMCs. The preliminary findings suggest that future studies are warranted, particularly in delineating whether these EEG features that differed between clusters simply reflect a spectrum of variation that, while potentially informative regarding mechanism, still entirely falls within normative values, or whether, for example, the reductions in stimulus onset and chirp processing responses in Clusters 1 and 2 are significantly decreased relative to non-PMC controls. Future studies with larger samples, enabling medication use and psychiatric comorbidities to be better disentangled, are also necessary to confirm these findings. Lastly, due to the focus on mood and anxiety, the sample was limited to female PMCs and lacks applicability to males with the premutation. Future work should aim to evaluate males only or include equal numbers to assess clinical profiles.

## 5. Conclusion

Overall, PMCs exhibited relatively typical electrophysiological measures at the group level. However, congruent with Schmitt et al. (2022), we find differences notable by cluster membership supporting initial cluster characterizations and conclusions that heterogenous symptom presentations reflect underlying spectrum characteristics forming unique subgroupings. Importantly, these neuropsychiatric groupings also reflect differences in basic neural sensory processing characteristics, suggesting a tie between basic synaptic function, sensory systems, and risk for specific neuropsychiatric phenotypes within individuals with the *FMR1* premutation.

## Data availability statement

The raw data supporting the conclusions of this article will be made available by the authors, without undue reservation.

## Ethics statement

The studies involving human participants were reviewed and approved by Cincinnati Children’s Hospital Medical Center. Written informed consent to participate in this study was provided by the participants’ legal guardian/next of kin.

## Author contributions

JN led all EEG data preprocessing, management, data analyses, and wrote the full manuscript. LS led all clinical data collection and analyses. LD consulted on data preprocessing and analyses. EP led all EEG data collection. CE and JS supervised all aspects of study design, funding, and participant recruitment. LE supervised all aspects of EEG experimental design, data analysis, and manuscript preparation. All authors contributed significantly to manuscript preparation.
